# Editorial: Integrating health: a One Health approach to antimicrobial resistance and sustainable practices

**DOI:** 10.3389/fmicb.2026.1993246

**Published:** 2026-09-22

**Authors:** Taru Singh, Lucas Carvalho Basilio Azevedo, Shashwat Singh

**Affiliations:** 1 Department of Medical Biotechnology, Amity Institute of Biotechnology, Amity University Noida, Noida, India; 2 Institute of Agricultural Sciences, Federal University of Uberlandia, Uberlândia, Brazil; 3 Department of Zoology, Dr. Harisingh Gour Central University Sagar, Sagar, India

**Keywords:** antimicrobial resistance, genomic surveillance, multidrug resistance, One Health, sustainable development

Antimicrobial resistance (AMR) is an increasingly complex global health challenge that cannot be addressed effectively by any one approach alone. The One Health approach identifies that human, animal, and environmental health are closely interconnected and therefore provides a framework for integrated action against the emergence and spread of antimicrobial resistance. Strong antimicrobial policies are particularly important for ensuring appropriate prescribing, restricting inappropriate over-the-counter antibiotic sales, and promoting responsible antimicrobial use in human health, veterinary medicine, and agriculture. Antimicrobial stewardship programs complement these measures by supporting appropriate prescribing through surveillance, education, clinical decision-making, and infection prevention. At the governance level, meaningful collaboration among healthcare professionals, veterinarians, agricultural stakeholders, regulators, researchers, and policymakers is essential for developing coordinated approaches to AMR monitoring and response. Taken together, these efforts provide the foundation for a sustainable One Health response that can preserve the effectiveness of antimicrobials and protect public health (Figure 1).

**Figure 1:**
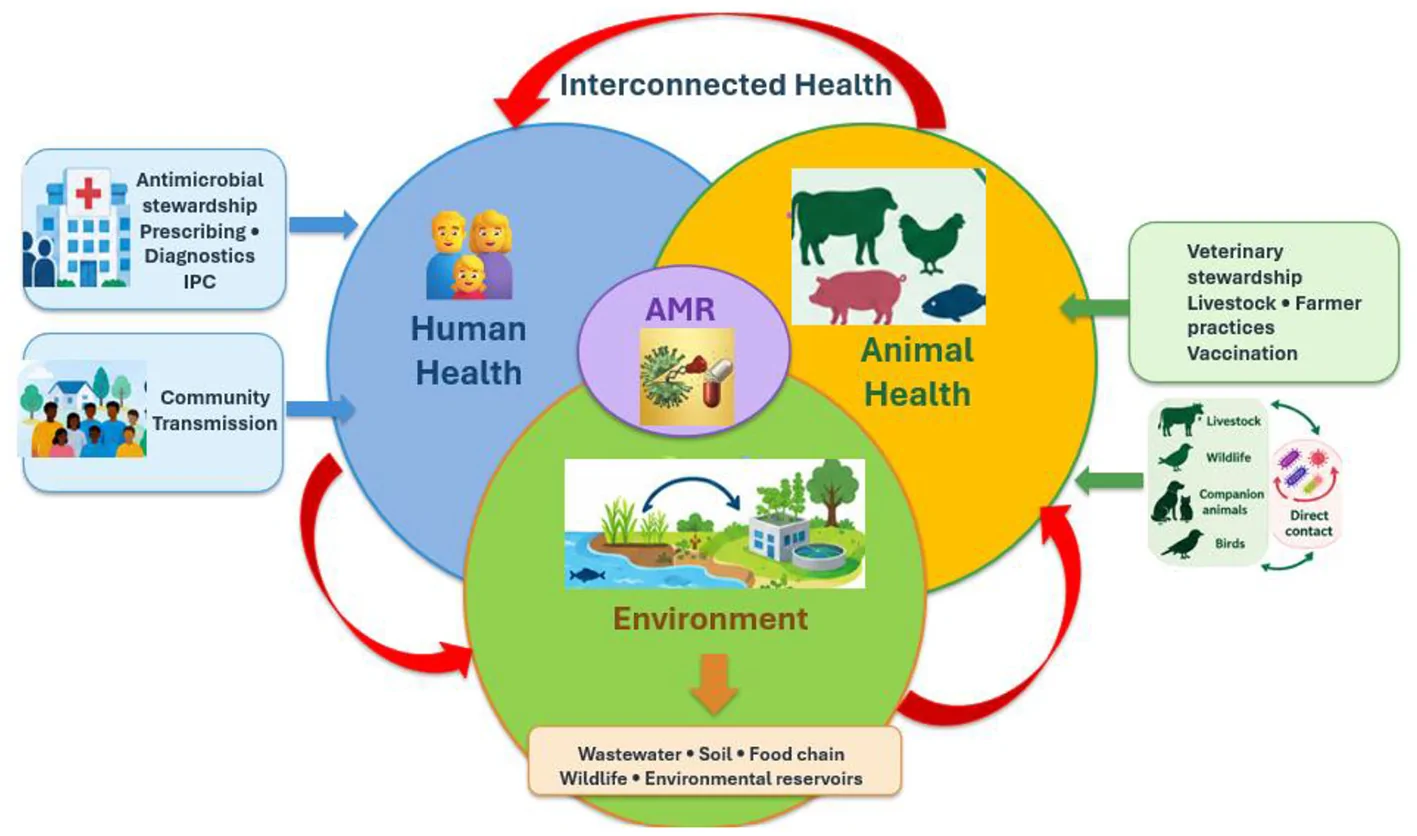
Integrated One Health framework for antimicrobial resistance, illustrating the interconnected drivers, transmission pathways, surveillance systems, prevention measures, stewardship activities, and sustainable interventions.

The survey by Prakoso and Rees, examined antibiotic decision-making for bovine respiratory disease among farm veterinarians in the UK, provides insight into the complexity of antimicrobial prescribing in livestock settings. Their findings show that prescribing decisions are influenced not only by clinical considerations but also by diagnostic uncertainty, animal welfare concerns, farmer expectations, economic pressures, and limited access to diagnostic facilities. Similarly, the work of Pereira et al. offers a useful framework for assessing veterinarians' knowledge, attitudes, and practices related to antibiotic use in animals. Such assessment tools can help to identify gaps in knowledge and behavioral factors that may contribute to inappropriate antimicrobial use.

Responsible antimicrobial use also depends on the strength of the systems in which prescribing decisions are made. Zavaleta-Monestel, Diaz-Madriz et al. identify antimicrobial consumption as an important indicator for assessing national AMR governance. Tracking antimicrobial consumption provides a measurable way of evaluating stewardship efforts and can complement existing surveillance and policy frameworks. In an associated contribution, Zavaleta-Monestel, Mosmann-Menezes et al. draw attention to the assumption that weaknesses in hospital antimicrobial stewardship programs are mainly the result of limited evidence, professional resistance, or technical barriers. Instead, they draw attention to structural and governance-related factors that can leave stewardship activities under-prioritized and treated as optional rather than as an essential part of patient safety. This perspective is important because even well-designed stewardship interventions are unlikely to succeed without institutional commitment. Stakeholder participation is equally important in antimicrobial stewardship programs. Using a participatory approach, Sow et al. show how participatory group model building can bring stakeholders together to better understand the drivers of antibiotic resistance and support the development of One Health policies.

Preventing infections is one of the most effective ways of reducing unnecessary antimicrobial exposure. Evidence from Dakhilallah et al., which compared different hand hygiene practices among nurses working in surgical settings of a Hospital, illustrates the continuing importance of appropriate hand hygiene in reducing microbial colonization. Although hand hygiene is a simple intervention, its consistent and evidence-based implementation remains fundamental to infection prevention. Extending this perspective, Rager and Scherenberg, examine the role of hand hygiene among cleaning staff in acute care hospitals. Their work highlights a group that is sometimes overlooked in IPC discussions but plays an important role in maintaining hygienic healthcare environments and limiting opportunities for pathogen transmission. A 6-year investigation by Lagadinou et al. provides a longitudinal perspective on vancomycin-resistant *Enterococcus* bloodstream infections over a 6-year period and considered the effects of the COVID-19 pandemic. Their findings illustrate how increased antimicrobial use, prolonged hospitalization, disruption of routine infection control practices, and pressure on healthcare systems can influence the epidemiology of resistant infections. The experience reinforces the need for IPC programs that remain functional and adaptable during periods of crisis, when healthcare systems are especially vulnerable to the emergence and transmission of resistant organisms.

Espín et al. draw attention to the fact that current surveillance remains heavily concentrated in clinical settings, while livestock, wildlife, food systems, wastewater, and other environmental reservoirs receive comparatively less attention. This imbalance can leave important transmission pathways undetected. Expanding surveillance across these interconnected environments would provide a more complete picture of how resistance emerges, persists, and spreads. Laboratory diagnosis remains an equally important component of surveillance. Through a nationwide proficiency study, Fernández-Cuenca et al., evaluate antimicrobial susceptibility reporting and carbapenemase detection in Gram-negative clinical isolates, revealing the difficulties laboratories may encounter when identifying complex resistance phenotypes. Their findings highlight the value of combining phenotypic susceptibility testing with molecular methods, particularly when confirmation of specific resistance mechanisms is clinically or epidemiologically important. In contrast, Sallam emphasizes that modifications to antimicrobial susceptibility breakpoints can substantially affect reported resistance rates. Consequently, an apparent increase or decrease in resistance does not always represent a true epidemiological change; it may partly reflect a change in the criteria used to interpret laboratory results. Consistent methodology and careful interpretation are therefore essential for generating reliable surveillance data that can inform public health decisions.

The spread of AMR is also closely linked to the movement of resistance genes between bacteria. Zhang, Li et al. draws attention to the importance of plasmids as mobile genetic elements capable of carrying and disseminating multiple resistance genes across different bacterial species. Their broad host range and ability to accumulate several resistance determinants make plasmids important drivers of multidrug resistance. For this reason, monitoring plasmid-mediated resistance should form part of broader AMR surveillance efforts. Another contribution by Zhang, Zhou et al. provides an example of horizontal gene transfer contributing to the emergence of carbapenem resistance in *Kluyvera ascorbata*, a species that is less commonly recognized as a clinically important reservoir. Such observations are a reminder that AMR surveillance should not focus exclusively on well-known pathogens. Less prominent bacterial species can also harbor clinically important resistance genes and potentially transfer them to more prevalent pathogens. Resistance surveillance should also extend beyond antibiotics. The work of Guo et al. examines bacterial adaptation to antiseptics commonly used in healthcare settings, drawing attention to the possibility of reduced susceptibility to agents that are central to infection prevention. Understanding how bacteria respond to antiseptic exposure is important for maintaining effective disinfection practices and for assessing whether such exposure may contribute to co-selection of antimicrobial resistance.

Geographical variation is further illustrated by Ramatla et al. who report considerable geographical variation in resistance patterns and emphasize the need for coordinated surveillance, stewardship, and new therapeutic approaches. Such geographical differences also illustrate how local antimicrobial use, healthcare practices, animal production, environmental contamination, and movement of organisms can interact to shape the resistance patterns. Wildlife represents another important but often underappreciated component, as Mbuthia and Hoza, identify wild birds as potential reservoirs of antimicrobial-resistant *Escherichia coli*. Because migratory birds move across national borders and between diverse habitats, they may contribute to the movement of resistant microorganisms between otherwise distant ecosystems.

Long-term AMR control will require strategies that reduce dependence on antibiotics rather than relying exclusively on the development of new antimicrobial drugs. One promising area is the use of microbiome-based approaches. Pattapulavar et al. explore the potential of probiotic-based therapeutics to reduce antibiotic dependence by supporting microbial homeostasis, improving colonization resistance, and influencing host immune responses. Their perspective moves beyond viewing probiotics simply as nutritional supplements and considers their potential role within broader One Health strategies.

Vaccination provides another well-established means of preventing infections before antibiotics are needed. Saleh et al. provide evidence of the impact of pneumococcal conjugate vaccination in the Eastern Mediterranean Region, where vaccination has reduced pneumococcal disease and may also contribute to lower antibiotic prescribing and reduced circulation of resistant pneumococcal strains. Vaccination therefore represents an important AMR intervention: by preventing infection, it can reduce antimicrobial consumption while also limiting opportunities for resistant organisms to emerge and spread. At the same time, preventive antimicrobial interventions must be evaluated carefully for their potential ecological consequences. Vanbaelen et al. examine the implications of doxycycline post-exposure prophylaxis (doxyPEP) for preventing selected bacterial sexually transmitted infections. Although doxyPEP can be effective in certain populations, its broader implications for antimicrobial resistance require continued monitoring. The potential benefits of preventing infection must therefore be considered alongside the possibility of increasing antimicrobial selection pressure and altering microbial communities.

We can conclude that AMR is a complex phenomenon that cannot be effectively controlled by a single discipline, intervention, or sector. Its drivers and consequences span human and animal health, agriculture, and the environment, making an integrated One Health approach essential. Effective action requires strong governance, responsible antimicrobial use, antimicrobial stewardship, infection prevention, improved diagnostics, genomic surveillance, education, vaccination, and continued research into sustainable alternatives. Such integration can help identify emerging threats earlier and allow interventions to be targeted where they are most needed.

